# Mechanisms of resistance to irreversible epidermal growth factor receptor tyrosine kinase inhibitors and therapeutic strategies in non-small cell lung cancer

**DOI:** 10.18632/oncotarget.21164

**Published:** 2017-09-22

**Authors:** Jing Xu, Jinghui Wang, Shucai Zhang

**Affiliations:** ^1^ Department of Medical Oncology, Beijing Tuberculosis and Thoracic Tumor Research Institute/Beijing Chest Hospital, Capital Medical University, Beijing, China

**Keywords:** NSCLC, irreversible EGFR-TKIs, resistance, mechanisms, therapeutic strategies

## Abstract

Epidermal growth factor receptor (EGFR) T790M mutation is the most frequent mechanism which accounts for about 60% of acquired resistance to first-generation EGFR tyrosine kinase inhibitors (TKIs) in non-small cell lung cancer (NSCLC) patients harboring EGFR activating mutations. Irreversible EGFR-TKIs which include the second-generation and third-generation EGFR-TKIs are developed to overcome T790M mediated resistance. The second-generation EGFR-TKIs inhibit the wide type (WT) EGFR combined with dose-limiting toxicity which limits its application in clinics, while the development of third-generation EGFR-TKIs brings inspiring efficacy either *in vitro* or *in vivo*. The acquired resistance, however, will also occur and limit their response. Understanding the mechanisms of resistance to irreversible EGFR-TKIs plays an important role in the choice of subsequent treatment. In this review, we show the currently known mechanisms of resistance which can be summarized as EGFR dependent and independent mechanisms and potential therapeutic strategies to irreversible EGFR-TKIs.

## INTRODUCTION

The epidermal growth factor receptor (EGFR) tyrosine kinase inhibitors (TKIs) significantly improve the outcomes as an initial treatment in non-small cell lung cancer (NSCLC) patients with activating EGFR mutations compared with standard platinum-doublet chemotherapy. However, acquired resistance will inevitably occur after initial treatment of EGFR-TKIs. It is reported that EGFR T790M mutation is the most frequent mechanism of acquired resistance, which accounts for about 60% and may be more common in patients with mutant EGFR L858R than EGFR 19del [1, 2]. The point mutation (ACG to ATG) in exon 20 leads to the replacement of threonine by methionine at position 790 which is a “gatekeeper” of EGFR to combine with ATP. This change mainly restores ATP affinity and decreases the combination of reversible EGFR-TKIs by the increased steric hindrance. To overcome the resistance caused by T790M, the irreversible EGFR-TKIs are developed.

Second-generation EGFR-TKIs is a quinazoline-based irreversible pan- human epidermal growth factor receptor (HER/ERBB) inhibitor. It has electrophilic functionality, covalently binding to cysteine residue of EGFR (afatinib to Cysteine-773 and dacomitinib to Cysteine-797) [3–5], which allows them to achieve a stronger bonding ability to ATP binding pocket than reversible EGFR-TKIs. In preclinical studies, afatinib and dacomitinib are effective to tumor harbouring T790M mutation and wide-type (WT) EGFR, although the IC50 is 10–500 fold higher than EGFR sensitive mutation alone [5–7]. But in NSCLC patients, they are only potent agents to mutant EGFR instead of EGFR T790M, because the suppression of WT EGFR leads to dose-limiting toxicity, such as skin rash/acne, paronychia, and diarrhea [8]. Therefore, researchers turned to the exploration of new agents.

The third-generation EGFR-TKIs, AZD9291, CO-1686, WZ4002, EGF816, and ASP8273 are irreversible small-molecule inhibitors being tested in clinical trials. Like other irreversible EGFR-TKIs, they irreversibly react with the cysteine-797 residue in ATP bonding pocket. But they are more specific in targeting EGFR T790M and EGFR activating mutation than WT EGFR, because the anilinopyrimidine scaffold can be better adapted to the conformation after T790M mutation and the binding force to T790M mutant is 100–200 times than WT EGFR. Thus they have greater clinical efficacy than second-generation EGFR-TKIs to inhibit EGFR T790M mutation [1, 5, 9]. The development of irreversible EGFR-TKIs brings inspiring efficacy either *in vitro* or *in vivo*. The acquired resistance, however, will still emerge and limit their response. Therefore, understanding the mechanisms of acquired resistance to irreversible EGFR-TKIs plays an important role in guiding subsequent treatment. We review the currently known mechanisms of resistance and potential therapeutic strategies to irreversible EGFR-TKIs.

### EGFR-dependent resistant mechanism

**Figure 1:**
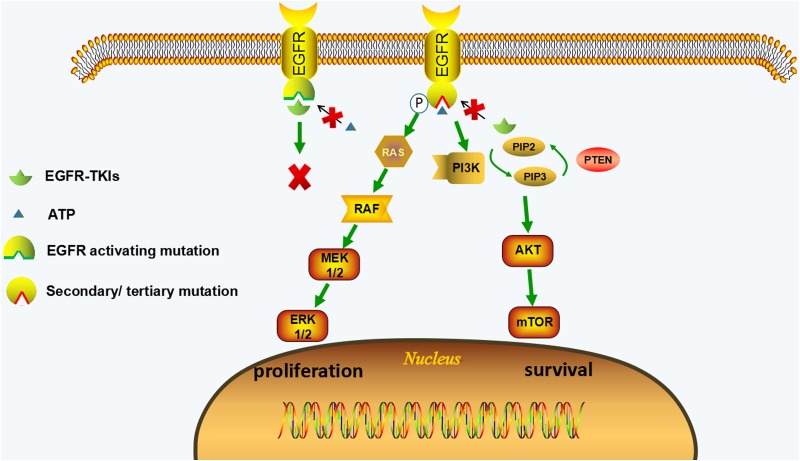
EGFR-dependent resistance mechanisms of irreversible EGFR-TKIs EGFR-TKIs compete with ATP for binding to tyrosine kinase domain of EGFR with activating mutations, leading to the inhibition of EGFR and its downstream pathways (MAPK and PI3K/AKT pathways). The secondary or tertiary mutations of EGFR (such as C797S mutation) can change the conformation of tyrosine kinase domain of EGFR, which hinder EGFR-TKIs from binding to EGFR and restore ATP affinity; therefore the downstream pathways are activated leading to the proliferative and anti-apoptotic effect. The activation of downstream signaling is coupled from upstream EGFR activation in EGFR-dependent resistant cells.

EGFR-dependent resistant mechanism is usually caused by secondary mutation in ATP-binding pocket of EGFR. The mutation can change the conformation of EGFR and thus the EGFR-TKI can no longer bind to EGFR. In EGFR-dependent resistant cells, the activated EGFR signaling is still playing the major role in promoting survival through its downstream signaling (Ras-MAPK signal and PI3K-AKT signal). Therefore, EGFR-targeted therapies should be potential approaches to this type of resistance. (Figure 1, Table 1)

**Table 1:** EGFR-dependent resistance mechanisms of irreversible EGFR-TKIs and potential treatment strategies

Resistant mechanisms	Drugs	Co-existed mechanism	Strategies	Reference
**2G-TKIs**				
T790M	HKI-272, afatinib	-	3G-TKIs Cetuximab plus afatinib	[10, 14, 15] [21–24]
T790M amplification	Dacomitinib	EGFR amplification	-	[17]
V843I	Afatinib, dacomitinib	-	-	[30, 31]
C797S	Afatinib, HKI-272	-	1G-TKIs	[29], [10]
C797S/G G796R/C/D L718A M766T T854A	Canertini(CI-1033)	-	-	[27]
E931G	CL-387,785	-	-	[28]
L792F	Afatinib	-	Dacomitinib	[29]
**3G-TKIs**				
C797S	AZD9291, WZ4002, CO-1686	-	Quinazoline based EGFR-TKIs	[32]
T790M/C797S	AZD9291, WZ4002 CO-1686, HM-61713	C-Met amplification,KRAS G12S mutation	3G-TKIs + 1G-TKIs (in trans allele) Chemotherapy (in cis allele) Cetuximab (L858R/T790M/C797S) EAI045+cetuximab (L858R/T790M/C797S) Brigatinib+ anti-EGFR antibody CUDC-101 (a EGFR/HER2/HDAC inhibitor) PKC412 (a broad spectrum protein kinase inhibitor)	[32–37, 39–42]
L718Q L844V	WZ4002, CO-1686	-	Quinazoline based EGFR-TKIs AZD9291	[32]
G796D	AZD9291	T790M loss	-	[169]
T790M/L718Q T790M/L844V	AZD9291, WZ4002, CO-1686	-	-	[32, 38]
T790M/L798I T790M/L692V T790M/E709K	CO-1686	-	-	[35]
T790M/C797G		EGFR amplification	-	[170]
T790M/L792F/Y/H, T790M/L718Q/V, T790M/G796S/R	AZD9291	T790M (in cis)/C797S (in trans)	-	[45], [43], [44]
T790M/G724S	EGF816	T790M loss	-	[61]
EGFR amplification	AZD9291, CO-1686	Src-AKT activation KRAS G13D EMT T790M/C797G,	CO-1686+Cetuximab Afatinib+Cetuximab	[35, 46–48]

“-”represents no co-existed mechanisms or no treatment strategies, 1G-TKIs: first-generation EGFR-TKIs; 2G-TKIs: second generation EGFR-TKIs; 3G-TKIs: third-generation EGFR-TKIs.

### EGFR-dependent resistant mechanism in second-generation EGFR-TKIs

### T790M mutation is a major mechanism of second-generation EGFR-TKIs

*In vitro*, the second-generation EGFR-TKIs can effectively inhibit EGFR T790M, thus researchers speculated whether these irreversible EGFR-TKIs could be superior to first-generation EGFR-TKIs in preventing or delaying the emergence of the resistance as a first-line therapy. In resistant cell lines derived from PC-9 cells (EGFR exon 19del) at clinically relevant concentration of neratinib (HKI-272), an EGFR/pan-HER inhibitor, EGFR T790M gatekeeper mutation was first revealed as an acquired resistance mechanism of NSCLC to second-generation EGFR-TKIs [10]. High dose of neratinib (~1 μmol/L) could cope with T790M mutation but the clinical application was hindered by dose-limiting toxicity. However, a phase 2 study had demonstrated that neratinib was not a potent inhibitor only with less than 3% of objective response rate (ORR) in EGFR-mutant NSCLC patients [11]. Dacomitinib and afatinib are proved to be more effective to NSCLC patients harboring EGFR activating mutation in clinical trials [8, 12, 13]. The emerging T790M mutation was also discovered as a resistance mechanism in afatinib resistant PC-9 cell lines even at high concentrations (1.5–2 μmol/L), and resistant degree of these cell lines was affected by gene dosage of the T790M allele although this phenomenon was failed to discovered in xenograft animal models [14, 15]. And besides, in cell lines with EGFR T790M mutation, the more resistant subclones harboring amplified T790M in cis with EGFR activating mutation would be selected leading to a more robust resistance under the treatment of high dose of dacomitinib [16, 17]. These pre-clinical studies indicate that the second-generation EGFR-TKIs cannot prevent the occurrence of resistance caused by T790M mutation irrespective of the drug concentrations.

T790M mutation was confirmed to be the resistance mechanism of second-generation EGFR-TKIs in tissue specimen. It is still the most important mechanism accounting for 30–50% of the resistance both in reversible EGFR-TKIs naive patients and treated patients according to the rencent studies. [18, 19]. (Table 2) But the phase II B lux-lung 7 (NCT01466660) study indicated that afatinib significantly prolonged the progression-free survival (PFS) compared with gifitinib (11.0 vs. 10.9 months; HR 0.73, 95% CI 0.57–0.95; *p* = 0.017) [13]. And the preliminary data presented at American Society of Clinical Oncology (ASCO) 2017 from a phase III ARCHER 1050 study (NCT01774721) also demonstrated dacomitinib was superior to gefitinib as the first-line treatment of NSCLC patients with mutant EGFR (PFS, 14.7 vs. 9.2 months; HR 0.59, 95% CI 0.47–0.74; *p* < 0.0001) [20]. These data may suggest second-generation EGFR-TKIs could delay the occurrence of resistance. It is worth comparing the different resistance mechanisms of afatinib or dacomitinib with gefitinib group to further investigate the underlying reasons of these results.

**Table 2:** The prevalence of important resistance mechanisms of second-generation EGFR-TKIs reported in recent studies

Agents	Study	Sample	Methods	Number	T790M mut % (*n*)	C-Met amp % (*n*)	Unkown % (*n*)	Multiple % (*n*)	References
Afatinib	Campo et al.	Tumor samples	SNaPshot FISH ICH	11	36.4% (4)	9.1% (1)	54.5% (6)	-	[18]
	Wu et al.	Tumor samples	Sequencing	42	47.6% (20)	NA	52,4% (22)	-	[19]

“-”represents there were no patients in the cohort; NA: not available; amp: amplification; FISH: Fluorescence *in situ* hybridization; ICH: Immunohistochemistry; SNaPshot: A multiplexed allele-specific PCR-based platform.

Patients with acquired resistance of T790M mutation to first/second-generation EGFR-TKIs exhibited good efficacy to third-generation TKIs in clinical trials [21, 22]. The recent AURA3 study (NCT02151981) [23] showed that AZD9291 significantly improved ORR and PFS compared with platinum/pemetrexed in T790M-positive NSCLC patients who had disease progression on first-line EGFR-TKIs. Cetuximab plus afatinib may be another therapeutic option to cope with T790M mediated resistance both in preclinical model and NSCLC patients. The combination therapy showed a dramatic efficacy in L858R/T790M model [24], while in clinics, the ORR was 32% and median PFS was 4.7 months (95% CI, 4.3–6.4) in T790M-positive patients (NCT01090011) [25].

### Some rare EGFR secondary mutation

According to a recent study, atypical mutations occur in 14% of NSCLC patients haboring EGFR-TKIs sensitizing mutation, the majority of which have nothing to do with drug sensitivity [26]. The atypical secondary mutation associated with drug resistance mainly locate in TKIs binding region or function area in kinase domain of EGFR, such as C797S/G mutations responsible for covalent interactions with second-generation TKIs; G796R/C/D mutation adjacent to Cys-797 position interfering with covalent interactions between EGFR and TKIs; L718A, M766T, T854A, L792F mutations which are mutations in important binding regions of TKIs; E931G mutation, which can affect the dimerization of EGFR and stop the signal transduction [10, 27–29]. Double mutation L858R/V843I can also cause resistance to second-generation EGFR-TKIs perhaps by increase of catalytic activity of EGFR [30, 31]. (Table 1)

### EGFR-dependent resistant mechanism in third-generation EGFR-TKIs

### C797S mutation

Cys-797 site in ATP binding pocket of EGFR is the one where irreversible EGFR-TKIs covalently bound to. Thus the point mutation of Cys797Ser located in exon 20 of EGFR results in acquired resistance to third-generation EGFR-TKIs. In cell lines and xenograft model with EGFR activating mutation (exon 19 deletion or L858R mutation) alone or EGFR activating mutation/T790M, the emerging of C797S caused drug resistant to AZD9291, CO-1686 and WZ4002 [32–34]. C797S mutation accounts for more than 19% of acquired resistance to AZD9291 while < 3% to CO-1686 in EGFR T790M mutant NSCLC patients previously treated with reversible EGFR-TKIs. (Table 3) And C797S mutation is more likely to happen in the patients with EGFR exon 19 deletion in current clinical resistant cases [34–38]. The evidences suggest that the resistance mechanism of third-generation EGFR-TKIs may vary by drugs and mutation types.

**Table 3:** The prevalence of important resistance mechanisms of third-generation EGFR-TKIs in T790M mutant patients reported in rencent studies

Agents	Study	Sample	Methods	Number	T790M mut %(n)	C-Met amp %(n)	Unkown %(n)	Multiple %(n)	References
Afatinib	Campo et al.	Tumor samples	SNaPshot FISH ICH	11	36.4%(4)	9.1%(1)	54.5%(6)	-	[18]
	Wu et al.	Tumor samples	Sequencing	42	47.6%(20)	NA	52,4%(22)	-	[19]

“-”represents there were no patients in the cohort; NA: not available; CAPP-Seq: Cancer personalized profiling by deep sequencing; DDPCR: Droplet Digital polymerase Chain Reaction; NGS: next-generation sequencing; ICH: Immunohistochemistry.

The emerging data shows the allocation pattern of T790M and C797S mutation may help the choice of treatment after acquire resistance based on C797S. In resistant cell lines, the double mutants (EGFR activating mutation/ C797S) were still sensitive to quinazoline-based EGFR-TKIs such as gefitinib and afatinib, while the cell lines harboring triple mutation (EGFR activating mutation/ T790M/ C797S) were resistant to all quinazoline-based EGFR inhibitors [32]. The resistance pattern of these triple mutant cells is further divided into cis-pattern and trans-pattern according to another research. When C797S and T790M are on separate alleles (trans-pattern), the resistant cells may be sensitive to the combined treatment of third- and first- generation EGFR-TKIs. While the two mutations are on the same alleles (cis-pattern), none of the existing EGFR-TKIs is effective [33]. According to the research, unlike EGFR L858R, the majority of EGFR activating mutations (EGFR exon 19 deletion, L858R/T790M and exon 20 insertion) could activate EGFR signal independent of asymmetric dimerization and thus were less sensitive to cetuximab [39]. However, cetuximab showed moderate effect on L858R/T790M/C797S mutant which activated EGFR signal partly by asymmetric dimerization. A newly developed allosteric inhibitor, EAI045 had an effect on resistance mouse model caused by L858R/T790M and L858R/T790M/C797S mutation together with cetuximab [40]. Moreover, brigatinib, a potent ALK inhibitor, was found to be active against cells harboring triple mutation since it could bind to the ATP-binding pocket of triple mutant EGFR, and the effect would be enhanced when combined with the anti-EGFR antibody [41]. Recently, Jacqulyne et al. reported that CUDC-101 (a EGFR/HER2/HDAC inhibitor) and PKC412 (a broad spectrum protein kinase inhibitor) could inhibit the cell lines with triple mutation at low concentrations, the non-covalent EGFR inhibitors might be new strategies to overcome resistance caused by C797S mutation [42].

### Other secondary/tertiary mutations of third-generation EGFR-TKIs

EGFR L718Q and L844V are another two drug resistance mutations which disrupt the covalent bond of inhibitor onto C797 [32, 38]. Unlike C797S, an in-vitro study indicated that the double mutants (EGFR sensitizing mutation/ L718Q or L844V) were only resistant to WZ4002 and CO-1686, while relatively sensitive to AZD9291 and gefitinib and afatinib [32]. But the triple mutants (EGFR sensitizing mutation/ T790M/ L718Q or L844V) were resistant to all EGFR-TKIs. EGFR T790M/L798I was a novel discovered mechanism leading to CO-1686 resistance which affected the binding stability of drug to EGFR by interfering with the formation of hydrogen bonding between Asp800 residues in EGFR and CO-1686 [35]. Bedides, G796S/R, L792F/Y/H mutations were reported as new AZD9291 resistance mutations which could coexist with C797S mutation on separate alleles. G796S/R mutation would occupy the position of aromatic ring on AZD9291 and keep the drug from binding to EGFR [43]. While L792F/Y/H mutation could sterically influence the methoxy group on the phenyl ring of AZD9291 and disable the positioning and binding of AZD9291 to EGFR [44, 45]. Other mutations associated with resistance are showed in Table 1.

### EGFR amplification

In CO-1686 resistant PC-9 cell lines, Nukaga et al. found EGFR WT allele was amplified. When expressed the WT EGFR in H1975 cell lines, they became resistant to CO-1686 or AZD9291 through activation of WT EGFR and downstream pathways induced by the ligand of EGFR [46]. The combination treatment of roclietinib or afatinib with cetuximab might be effective to this WT dependent resistance. EGFR amplification was detected in 9~25% of CO-1686 resistant patients [35, 47] and 4~35% of AZD9291 resistant patients with mutant T790M [48, 49]. (Table 3)

### EGFR- independent resistant mechanism

**Figure 2:**
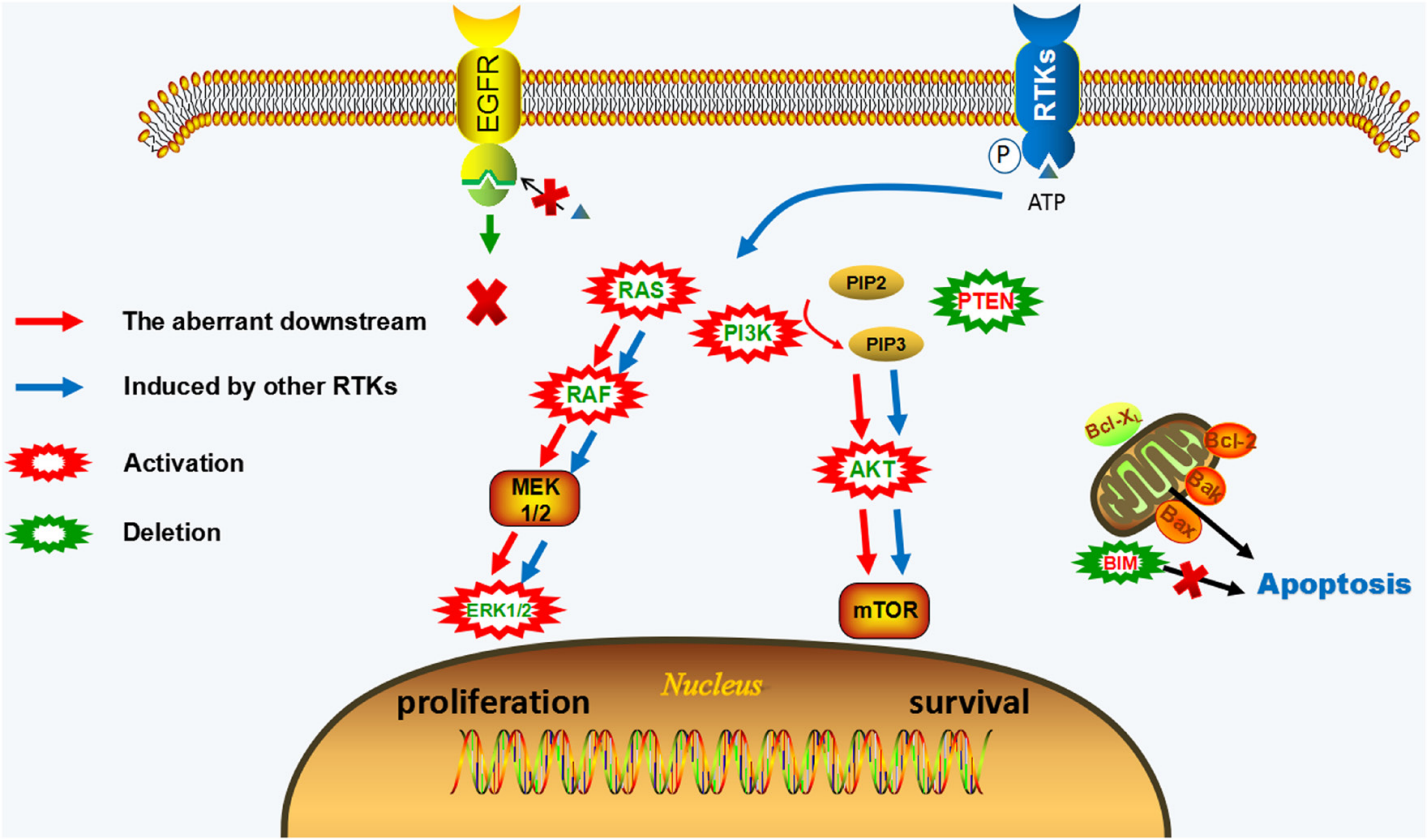
EGFR-independent resistance mechanisms of irreversible EGFR-TKIs EGFR phosphorylation still can be blocked by EGFR-TKIs, but downstream pathways of EGFR remain activated. The activation of other RTKs (c-Met, HER2, IGF1R, FGFR1, etc.) or the aberrant activation of downstream pathways (KRAS gain/mutation, NRAS gain/mutation, BRAF mutation, MAPK1 amplification, PIK3CA mutation, AKT3 activation, PTEN deletion) offer a way bypass EGFR to reactivate of MAPK and PI3K-AKT signaling leading to the proliferative and anti-apoptotic effect. The activation of downstream signaling is uncoupled from upstream EGFR activation in EGFR-independent resistant cells.

When EGFR independent resistance occurs, EGFR phosphorylation still can be shut down by inhibitors, but the resistant cells acquire an alternative way for survival and proliferation. EGFR-independent mechanisms of irreversible EGFR-TKIs mainly include the activation of other parallel signaling, the aberrant downstream pathways, and phenotypic transformation. The combined therapy targeting these resistance mechanisms with EGFR-TKIs may be potential treatment strategies. (Figure 2, Supplementary Table 1)

### The activation of alternative signaling

The activation of alternative receptor tyrosine kinases (RTKs) signaling reactivates mitogen-activated protein kinases (MAPK) and phosphatidylinositol-3-kinase (PI3K)/ protein kinase B (AKT) downstream pathways regardless of the inhibited EGFR. Consequently, the dependence of these resistant cells on EGFR for survival and proliferation is weakened or disappeared. The interactive cross-talk between EGFR and other RTKs such as c-Met, HER2, and IGF1R may be an underlying event mediating EGFR-TKIs resistance based on this kinase switch [50, 51].

### HGF/MET signaling

Human mesenchymal-epithelial transition factor (c-Met) belongs to RTK superfamily whose ligand is hepatocyte growth factor (HGF) [52]. C-Met amplification is a frequent event accounts for 5–22% of acquired resistance to reversible EGFR-TKIs in NSCLC patients [53–55] and can exist concurrently with T790M mutation. From in-vitro studies, c-Met amplification activated the ErBB3-PI3K-AKT signaling, which led to EGFR-TKIs resistance by allowing the cells independent of EGFR signaling [53, 54, 56]. HGF overexpression is also frequently detected in about 61% acquired resistant patients of EGFR-TKIs [57], but unlike c-Met amplification, it induces TKI resistance by activating Met-PI3K-AKT axis but has nothing to do with ErBB3 [58].

Met signaling provides an alternative route bypassing EGFR which can limit the activity of irreversible EGFR-TKIs in EGFR-mutant NSCLC. In-vitro studies showed that c-Met inhibitor (SU11274 or crizotinib) could improve the sensitivity of H1975 (EGFR L858R/ T790M) and HCC827ER (EGFR exon 19 del/c-Met amplification) cell lines to irreversible EGFR-TKIs (afatinib and WZ4002) even when HGF was present [59, 60]. When blocking c-Met and EGFR together, the MAPK and PI3K-AKT signaling were down-regulated, thus induced the enhanced proliferation inhibition and apoptosis. In EGFR mutant NSCLC patients, c-Met amplification with mutant or WT T790M before treatment can limit the response to CO-1686. These results suggest that c-Met signaling contribute to primary TKIs resistance in WT and mutant T790M cell lines. Met signaling also mediates acquired resistance to irreversible EGFR-TKIs. In a small study of NSCLC patients with sensitizing EGFR mutation, c-Met amplification was detected in 9% of repeat biopsies after progression of first-line afatinib treatment [18]. (Table 2) And recent studies showed that c-Met amplification might be a frequent mechanism detected in 5~30% of post-progression specimens from EGFR T790M positive patients treated with the third-generation EGFR-TKIs [35, 49, 61].(Table 3) Besides, the coexistence of multiple resistance mechanism is a common phenomenon. In afatinib resistant cell lines with EGFR T790M mutation, triple targeting of ERBB3 + c-KIT + c-Met by siRNA could kill more than 70% of afatinib resistant H1975 clones [62]. This indicates that c-Met can occur with other resistance mechanism. They mediate acquired resistance of afatinib together by providing another bypass way for cell survival. C-Met can also co-occur with ERBB2 amplification or EGFR C797S mutation in NSCLC patients that acquire resistance to AZD9291 or CO-1686 according to recent reports [35, 36].

HGF, the ligand of c-Met, can also induce resistance to irreversible EGFR-TKIs. When HCC827 and PC-9 (EGFR exon 19 deletions) or H1975 (EGFR L858R/T790M) cells were co-cultured with MRC-5 cells (human embryonic lung-derived fibroblast cell line that secreted HGF), the cell lines became resistant to irreversible EGFR-TKIs (CL-387,785, afatinib and WZ4002) mediated by high levels of HGF in a paracrine manner [60, 63]. In clinics, high serum HGF level before treatment was reported to be associated with poor outcomes of NSCLC patients treated with EGFR-TKIs, including afatinib [64–66]. HGF leads to drug resistance mainly through two mechanisms: HGF can accelerate the development of Met amplification [67] and increase the ties between EGFR and c-Met which has been disrupted by EGFR-TKIs [63]. As a result, the presence of HGF can activate downstream pathways though c-Met instead of EGFR.

Met kinase inhibitor plus EGFR-TKIs is a potential therapy to overcome TKIs resistance induced by the abnormity of Met signaling. In preclinical studies, SGX-523 (a selective c-Met inhibitor) [56, 68], crizotinib (an ALK/c-Met/ROS-1 inhibitor) [56, 60, 68], PHA-665752 (a c-Met kinase inhibitor) [69], E7050 (c-Met/VEGFR-2 inhibitor) [70], JNJ-61186372 (an EGFR/Met antibody) [71], tepotinib (a selective c-Met inhibitor) [72], anti-HGF neutralizing antibody, and HGF antagonist NK4 [63] plus new generation EGFR-TKIs can lead to dramatically regression of resistant tumor and delay the occurrence of drug resistance. Met inhibitor (crizotinib) monotherapy might be also feasible in disease control and symptom improvement when EGFR T790M was observed at a low level based on a case report [73]. The combined therapy of AZD6094 (a met inhibitor) and AZD9291 is being tested in c-Met positive patients with a progressive disease after third-generation EGFR-TKIs in the ongoing TATTON study (NCT02143466).

### IGF signaling

Insulin-like growth factor (IGF) signaling consists of its ligands (insulin, IGF-1 and IGF-2), receptors (insulin receptor, IGF1 receptor and IGF2 receptor) and IGF binding proteins (IGFBPs) [74]. IGF1 receptor (IGF1R) is an tyrosine kinase receptor which can be activated by IGF-1, IGF-2 or insulin [74]. When the ligands bind to IGF1R, the two important downstream pathways—MAPK signaling and PI3K-AKT signaling are activated and participate in cell proliferation and anti-apoptosis in cancers [75]. In preclinical studies, IGF1R activation mediated the acquisition of resistance to dacomitinib and WZ4002 mainly through activation of PI3K-AKT pathway in EGFR-mutant NSCLC cells, while MAPK pathway was activated subsequently only when prolonged the drug exposure [76]. But IGF signaling does not always lead to EGFR-TKIs resistance depending on PI3K-AKT pathway. In cell lines harboring T790M mutation, the PI3K-AKT and MAPK pathways were induced simultaneously by the activation IGF1R signaling responsible for afatinib [77] and WZ4002 [78] resistance. IGF binding protein 3 (IGFBP3) is also associated with EGFR-TKIs resistance. It usually functions as a negative regulator of IGF1R signaling by binding to the ligands of IGF1R [79], so the downregulation of IGFBP3 is related to drug resistance [76]. But it was also reported that the increased IGFBP3 level could potentiate the bioactivity of IGF and cause afatinib resistance by activation of IGF1R in PC-9 cell lines [80]. The research indicated that metabolites of CO-1686 had potency against IGF1R, which could cause hyperglycemia of patients [81] . While on the other hand, it will also address the question of whether CO-1686 can prevent the occurrence of IGF1R activation as an acquired resistant mechanism. So far, IGF1R activation is not reported in the patients who have disease progression after irreversible EGFR-TKIs.

IGF inhibitors, such as BMS536924, recombinant IGFBP3 [76], BI836845, linsitinib [77, 80], and AG-1024 [78] can restore the sensitivity to EGFR-TKIs based on the in-vitro and *vivo* study. But only the combination therapy of IGF1R and EGFR inhibition is demonstrated to be effective in preclinical studies, which perhaps because of the disruption of the existing crosstalk between EGFR and IGF1R or other RTKs [77]. An ongoing clinical trial (NCT02191891) is being conducted to assess the efficacy of afatinib plus BI836845 in mutant EGFR patients with progression disease after prior EGFR-TKIs treatment.

### HER2 signaling

HER2 is a member of ERBB family which lack its specific ligand so that it only can form heterodimers with other ERBB receptors [82]. EGFR is one of its important partners and they form a robust complex to activate downstream pathways [83]. Since the crosstalk between EGFR and HER2, researchers speculate that HER2 genomic gains and overexpression may be correlated with better response to EGFR-TKIs, which is supported by several study in TKIs naive NSCLC patients with positive EGFR expression [84–86]. However, HER2 amplification was also correlated with acquired resistance to EGFR-TKIs and detected in about 12% of the EGFR-mutant patients developed acquired resistance to first-generation EGFR-TKIs [87, 88]. Knockdown of HER2 in the cell line with EGFR 19del/T790M mutation partly restored sensitivity to afatinib, while introducing WT HER2 did not cause resistance to afatinib but erlotinib in EGFR mutant cell lines [87]. Thus, whether HER2 amplification is a resistance mechanism of second-generation EGFR-TKIs remains further investigation. HER2 amplification also contributed to CO-1686 and AZD9291 resistance [35, 36, 89]. Pre-existing HER2 amplification was associated with poor response to CO-1686, which suggested it might be a primary resistance mechanism of third-generation EGFR-TKIs. HER2 amplification was also found in less than 10% of T790M mutant patients whose disease had progressed after CO-1686 or AZD9291 treatment. (Table 3) It can co-occur with EGFR T790M mutation, c-Met or EGFR amplification as an acquired resistance mechanism of third-generation EGFR-TKIs [35, 36]. With regard to the influence of HER2 on sensitivity to EGFR-TKIs, Cappuzzo et al explained that the increasing cooperation between EGFR and HER2 led to better response to TKIs when the presence of increased copy number of HER2, while the high level of HER2 amplification made the tumor become more dependent on HER2 than EGFR signaling and therefore led to drug resistance [90].

The second-generation EGFR-TKIs such as afatinib, dacomitinib, and neratinib were proved to be potent inhibitors in blocking HER2 signaling *in vitro* and *vivo* studies [3, 91, 92]. While in clinical studies, these inhibitors were effective against mutant-HER2 but might have a poor response in amplified HER2 patients [93]. It was reported the ORR was 50% and disease control rate (DCR) was 100% for afatinib after first-line chemotherapy [93]. But in a phase II clinical study (NCT00818441), dacomitinib had variable efficacy in HER2 driven NSCLC patients with 12% and 0% of ORR in HER2 mutation and amplification cohort respectively [94]. Besides, trastuzumab which was approved for the treatment of breast cancer with HER2 overexpression only had a modest effect in lung cancer patients with HER2 amplification and HER2 overexpression [95, 96]. But trastuzumab combined with paclitaxel are currently assessing in a phase II clinical study (NCT02226757) as a treatment strategy in EGFR-mutant NSCLC patients developed HER2 activation (HER2 immunohistochemistry (IHC) ≥ 1) as acquired resistance mechanism to EGFR-TKIs. The combination was effective and well tolerated (ORR, 41%; median duration of response, 9 months) according to the latest result [97]. Furthermore, an in-vitro study suggested that trastuzumab emtansine (T-DM1) which was composed of microtubule polymerization inhibitor DM1 and trastuzumab could cope with gefitinib resistance caused by HER2 amplification [98]. HER2 amplification may be a common event in patients with acquired resistance to third-generation EGFR-TKIs, thus anti-HER2 therapy may be a promising area of research.

### Other alternative signaling

### Gas6/AXL activation

Growth arrest specific 6 (Gas6)/ anexelekto (AXL) activation is a kinase switch resistant mechanism of EGFR-TKIs. This resistance is often caused by increased expression of Gas6/AXL at RNA or protein level, while lack the genetic alteration, such as AXL mutation or AXL amplification [9, 48, 99]. Gas6/AXL activation was often accompanied by epithelial-mesenchymal transition (EMT) which was observed in CO-1686 resistant H1975 cell lines and AXL inhibition could restore the CO-1686 sensitivity [9].

### FGFR1 activation

Fibroblast growth factor receptor 1 (FGFR1) activation mediated acquired resistance to afatinib and AZD9291 *in vitro*. In afatinib resistant PC-9 cells and AZD9291 resistant HCC4006 cells, FGFR1 was activated by its ligand FGF2 through autocrine fasion and the resistant cell lines were sensitive to FGFR inhibitors, such as PD173074, axitinib and BGJ398 [48, 100]. In clinics, focal FGFR1 amplification was found in about 4% of AZD9291-resistant patients and often together with T790M loss [48, 49, 101].

### IL-6 activation

In an in-vitro study, activation of Interleukin 6 receptor (IL-6R)/ Janus kinase 1 (JAK1)/ Signal transducer and activator of transcription 3 (STAT3) mediated de novo resistance to afatinib and dacomitinib through IL-6 autocrine or paracrine loop in T790M mutant cell lines. Increased sensitivity to afatinib was observed when blocking IL-6R/JAK1/STAT3 signaling [102].

### SFK/FAK activation

The phosphorylation of Src family kinases (SFK) and focal adhesion kinase (FAK) were increased after AZD9291 treatment and SFK inhibitor (PP2, dasatinib, bosutinib and saracatinib) and FAK inhibitor (PF573228) could improve the proliferation inhibition of AZD9291. Besides, amplification of YES1, a SFK family member, contributed to acquired resistance to AZD9291 in T790M mutant cell lines [103]. Recently, Fan et al. reported that YES1 amplification was related to resistance to all three generations of EGFR-TKIs and identified in 4% of patients following progression on EGFR-TKIs [104].

### EPHA2 overexpressed

*In vitro*, Amato et al. found that erlotinib resistant PC-9 and HCC827 cell lines depended on EPH receptor A2 (EPHA2) signaling for survival. The overexpression of EPHA2, a kind of transmembrane glycoprotein with RTK activity, mediated acquired resistance to erlotinib and EPHA2 inhibitor could inhibit cell viability in erlotinib resistant cell lines. They also found that blocking EPHA2 signaling by ALW-II-41-27 could inhibit the survival of AZD9291 resistant cells, indicating that EPHA2 played a role in AZD9291 resistance [105].

### The aberrant downstream pathways

MAPK and PI3K/AKT are two important downstream pathways of EGFR. MAPK pathway is mainly correlated with cell proliferation in tumor, while the AKT signaling is mainly involved in anti-apoptotic regulation [106]. The alterations of critical components in downstream pathways result in aberrant activation of MAPK and PI3K/AKT signaling which are not under the control of EGFR, thus the drug resistance to EGFR-TKIs finally occurs.

### The alterations in MAPK pathway

RAS-RAF-MEK1/2-ERK1/2 signaling is one of the most characteristic pathways in MAPK pathways [107]. RAS (KRAS, NRAS and HRAS), RAF (ARAF, BRAF and CRAF) and MEK1/2 genes encode the key proteins in MAPK pathway and the alterations of these genes can usually lead to dysregulation of MAPK pathways.

### KRAS alteration

KRAS mutation frequently occurs in NSCLC accounting for 15–30% and correlate with primary resistance to EGFR-TKIs [108]. In general, mutant KRAS and EGFR are two mutually exclusive genes only found in < 2% cases of NSCLC patients [109–112]. Researchers believe that this co-mutation may have toxicity to cell viability, while the effect is decreased when EGFR signaling is blocked by EGFR-TKIs [36, 113]. As a result, KRAS mutation appears in EGFR-mutant cells after prolonged exposure to EGFR-TKIs as an acquired resistance mechanism [114]. Nevertheless, KRAS mutation was only revealed in a mouse model but not found in tissue samples of patients as an acquired resistance mechanism of first- and second- generation EGFR-TKIs [19, 55, 115, 116], which might be largely attributed to intratumoural heterogeneity. Recently, liquid biopsy overcomes this problem. According to a study analyzing cell-free circulating tumor DNA (cftDNA) based on droplet digital polymerase chain reaction (DDPCR) platform, KRAS mutation at codon 12 was found in 16 out of 33 (48.5%) plasma samples from patients after EGFR-TKIs progression and 13 (39.4%) had the commutation of KRAS and T790M [2]. Furthermore, KRAS mutations (KRAS G12A/S, Q61H/K, A146T and G13D) were detected in plasma samples of CO-1686 or AZD9291 resistant patients with mutant T790M , which ranged from 5–14%. [35, 36, 45, 101]. The emergence of KRAS mutation (KRAS G12S) was also reported in mutant T790M biopsy tissue at relapse under AZD9291 by targeted sequencing and the mutant KRAS transduced cells (PC-9 or HCC827) were less sensitive to third-generation TKIs compared with WT KRAS transduced cells [36].

### NRAS alteration

NRAS mutation is found only in ~1% [117–119] of NSCLC patients. Although NRAS alterations were not found in tissue samples collected after acquiring resistance to EGFR-TKIs [19, 88, 116], They (NRAS amplification, NRAS Q61K, E63K, G12V and G12R mutation) were confirmed to be associated with acquired resistance to EGFR-TKIs in pre-clinical data. NRAS Q61K mutation was first found in gefitinib resistant PC-9 cells [120], then NRAS E63K, Q61K, G12V and G12R mutation as well as WT NRAS gain were identified in AZD9291, WZ4002 and ASP8273 resistant cells with activating EGFR mutation or EGFR T790M mutation. NRAS gene alterations were not rare in EGFR-TKIs resistance especially in WZ4002 or AZD9291 resistant cell lines according to this preclinical study [15, 68].

### BRAF alteration

BRAF mutation is only described in ~5% of NSCLC patients [121–123]. One recent study reported that the prevalence of BRAF mutation was 7% in NSCLC patients with mutant EGFR and these patients still benefited from gefitinib treatment [109]. BRAF mutations (V600E and G469A) were found only in 1% of acquired resistance patients received first-generation EGFR-TKIs and also can co-existed with EGFR T790M mutation [116]. Based on a case report, BRAF V600E was detected in a patient progressed on AZD9291 treatment and the cells were derived from malignant pleura effusion (MPE), it can be suppressed by the combination of BRAF inhibitor (encorafenib) and AZD9291 [124]. A rencent study presented at ASCO 2017 showed that BRAF V600E was detected in 10% (2/21) of EGFR T790M mutant NSCLC patients with acquired resistance treated with AZD9291 [101]. BRAF rearrangement was also associated with acquired resistance and found in 25% (2/8) of post EGF816 progression samples [61]. So far, there is no report about BRAF mutation in acquired resistance to second-generation EGFR-TKIs yet [19].

### Other alteration in MAPK cascades

Other resistance mechanisms of EGFR-TKIs depending on MAPK signaling are also demonstrated to be associated with resistance to irreversible EGFR-TKIs. MAPK1 (EKR2) amplification and the decreased of negative regulators in MAPK signaling such as dual specific phosphatase 6 (DUSP6) led to WZ4002 resistance [15, 76, 125, 126]. CRKL (encode Crk-like protein and join in MAPK signal transduction as an adapter protein) amplification conferred to AZD9291 resistance [15, 114] and downregulation of neurofibromin (NF1), a negative regulator of MAPK pathway, was associated with primary and acquired afatinib resistance [127].

The combination of irreversible EGFR-TKIs with MEK inhibitor (selumetinib or trametinib) could overcome or delay the emergence of acquired resistance based on the activation of MAPK according to the in-vitro study [128]. An in-vitro study showed that KRAS gain played an important role in acquired resistance to EGFR-TKIs (gefitinib, afatinib, WZ4002, or AZD9291), but the resistant cells were only sensitive to the combined treatment when the KRAS expression was at low dosage [15]. Based on these inspiring results, there is an urgent need to develop biomarkers to predict the efficacy of MEK inhibitors. Despite the activation of molecules (MEK or ERK) in MAPK pathway, the basal levels of phosphorylated and total protein are not able to predict the sensitivity.

### The alterations in PI3K-AKT pathway

PI3K, AKT (also called protein kinase B) and PTEN are the important components in PI3K-AKT pathway. The activated PI3K can catalyse phosphorylation of PIP2 into PIP3, while phosphatase and tensin homologue deleted on chromosome ten (PTEN) plays a negative role in this process [129]. PIP3 bind to AKT as a critical second messenger, then AKT is activated through itself phosphorylation [130]. Once activated, various downstream kinases like mammalian target of rapamycin (mTOR) are triggered to perform different biological functions [131]. The abnormalities of these protein lead to high level PI3K-AKT pathway activation uncoupled from upstream EGFR phosphorylation, which mediate EGFR-TKIs resistance.

### PIK3CA mutation

PIK3CA encode p110α subunit which has catalytic activity in PI3K, thus the alteration of PIK3CA including mutation, amplification and over-expression would lead to activation of PI3K-AKT pathway [130, 132]. Whether PI3KCA alteration is a primary resistant mechanism of EGFR-TKIs remains unclear because of the controversial conclusions according to several studies [109, 133–137], but it is certain in acquired resistance to first-generation EGFR-TKIs accounting for ~5% of patients after disease progression and can coexist with T790M [136, 138]. As for irreversible EGFR-TKIs, PIK3CA mutation is a frequent event in acquired resistant to CO-1686 occurring in 12% (5/43) of EGFR T790M positive patients according to a study [35]. It was also associated with AZD9291 resistance and found in about 4% of post-progression samples of T790M mutant NSCLC patients [49, 101]. However, so far, there are no reports about the other irreversible EGFR-TKIs resistance based on PIK3CA alteration [19, 34], While in a case report, a patient with EGFR L858R/T790M/PIK3CA mutation after progressing on erlotinib did not response to afatinib plus cetuximab treatment which was effective in preclinical model and NSCLC patients with T790M mutation. Thus PIK3CA mutation is speculated to play a role in afatinib resistance which calls for further study [139].

### PTEN deletion

PTEN deletion [140] and decreased PTEN expression [141, 142] were proved to be connected with acquired resistance to first-generation EGFR-TKIs *in vitro* study. The decreased PTEN expression was detected in 1 out of 10 tumor samples from NSCLC patients with progression disease after gefitinib treatment [142]. While PTEN deletion might be a rare resistant mechanism only found in one EGFR 19del/T790M mutant case progressed on AZD9291. The proportion of PTEN deletion and EGF expression were higher in her AZD9291 resistant biopsy than baseline biopsy, which indicated these two alterations might contribute to AZD9291 resistance [48].

### AKT activation

There are three AKT isoforms which express in the majority of tissues—AKT1, AKT2, AKT3. A recent preclinical study showed that the expression of AKT3 and AKT2 were up-regulated in CO-1686 resistant H1975 cell lines compared with parental cell lines, while no increase in AKT1 [9]. AKT inhibitors MK-2206 and GDC-0068 could partly restore the sensitivity to CO-1686 which demonstrated that the activation of AKT play a role in CO-1686 resistance.

### Histologic transformation

### Epithelial-mesenchymal transition (EMT)

EMT is a biological process that cells with epithelial phenotype are converted into the mesenchymal phenotype and acquire the ability of migration [143]. The morphological changes of EMT cells are embodied as the loss of adhesion between cells-cells and cells-extracellular matrices (ECMs), the disorganized structure of cells and the disappearance of polarization [144]. Meanwhile, molecule changes also take place, such as the loss of proteins associated with cell junctions like E-cadherin and claudins, and up-regulation of mesenchymal markers like vimentin and fibronectin [145]. As a result, the mesenchymal-like cells acquire a higher migration potential, which is the most important symbol of EMT and therefore plays an role in tumor progression [146].

As an acquired resistance mechanism of EGFR-TKIs, EMT was firstly revealed in a study from an analysis of tumor biopsies of 37 NSCLC patients who developed resistance to EGFR-TKIs. It was an independent mechanism having no overlap with others. In order to figure out how EMT occurs and leads to drug resistance, some vitro studies are carried out. Transforming Growth Factor (TGF)-β and other contextual signals such as Wnt and Notch signal which epithelial cells receive from their microenvironment may induce and maintain this phenotype change [143, 144, 147, 148]. These extracellular signals will activate many cellular signal transduction pathways (such as smad signal pathway) and then induce or suppress the expression of many genes. In a recent study, a 76-gene EMT signature was developed to predict the resistance to first-generation EGFR-TKIs. The changes were related to molecule events in genetic and epigenetic levels of the four EMT markers (CDH1, VIM, CDH2, and FN1) and might be the key point in EMT [149]. Therefore, EGFR-TKIs resistant cells do not depend on EGFR any more, on the contrary, the resistant cells rely on other network associated with mesenchymal phenotype.

EMT has been proved to be a mechanism of acquired resistant to irreversible EGFR-TKIs. In the afatinib-resistant/afatinib-crizotinib-resistant HCC827 cell lines (AR/ACR) and afatinib-resistant HCC4006 cell lines, EMT was observed. In addition to the downregulation of E-cadherin and upregulation of vimentin, micro RNA-200c, a negative regulator of zinc finger E-Box binding homeobox 1 (ZEB1), was downregulated and modified in these resistant cell lines [150]. The majority of the EMT cell lines had stem cell-like properties expressing putative stem cell markers (ALDH1A1, ABCB1, etc.) and showing docetaxel resistance. Similarly, resistant cells to third-generation EGFR-TKIs also exhibit signs of EMT and are independent of EGFR signal. According to a report, Gas6/AXL signal was activated in CO-1686 resistant NCI-H1975 cell lines (COR) and might stimulate the PI3K-AKT signal to promote cell survival [9]. The enhanced autophagy is another way of resistant cells surviving. The WZ4002 resistant NCI-1975 cell lines (WR) relied upon autophagy and Pin1 activity for survival and proliferation [151]. Another signal pathway associated with EMT—NF-κB was found in CNX-2006-resistant cell lines (CR), but the researchers did not think the EMT as a key role of resistance [152].

The EMT may not be a pan-resistance character; instead, these resistant cells exhibit sensitivity to other agents. Researches showed that the cells with mesenchyma phenotype were resistant to common cytotoxic chemotherapies, such as docetaxel [150], pemetrexed [149], but relative sensitive to cisplatin, gemcitabine, etoposide and vinorelbine [144, 149]. These results indicate that the use of some chemotherapeutic agents may be a better choice for EGFR-TKIs resistance undergoing EMT. Some potential inhibitor targeting specific molecules of EMT signal can also restore the sensitivity to irreversible EGFR-TKIs. (Supplementary Table 1)

### Small cell lung cancer transformation

The small cell lung cancer (SCLC) transformation is observed in ~14% of the re-biopsy tumors from patients after resistance to first-generation EGFR-TKIs [55, 153, 154]. There are two hypotheses about the small cell lung cancer transformation. One is tumor heterogeneity that the pre-existing SCLC components are selected under TKIs treatment. Another is the occurrence of molecule events which lead to adenocarcinoma transforming to SCLC [155]. The inactivation of Tp53 and Retinoblastoma 1 (Rb1), PIK3CA mutation and lose EGFR expression are common molecule events in SCLC transformation [55, 156]. Niederst et al. revealed that the loss of RB1 was detected in 100% of SCLC transformed biopsy samples or cell lines [156]. Oser et al. explained that these genetic events promoted the occurrence of SCLC transformation. EGFR signaling was able to drive alveolar type II cells to differentiate into adenocarcinoma, while EGFR-TKIs could block this process. Once these molecule events occurred, alveolar type II cells would differentiate into SCLC [155].

In a cohort of T790M mutant patients who had developed resistance to CO-1686, about 17% (2/12) patients underwent SCLC transformation. Both of them retained EGFR activating mutation and lost RB [47]. SCLC transformation was also detected in about 4% of patients that developed resistance to AZD9291 [48, 49, 101]. Interestingly, the majority of these cases were accompanied by T790M loss, the links between this two events required further investigation. The combination chemotherapy of etoposide and platinum may be a treatment option for the patients with SCLC transformation [157].

### Other EGFR-independent mechanisms

### T790M loss

T790M loss may be a common acquired resistant mechanism of third-generation TKIs, rencent studies showed that about half of T790M-mutant patients developed resistance to third-generation TKIs due to T790M loss [34, 45, 47, 49, 101]. This mechanism is most probably associated with selective pressure of continuous therapy and heterogeneity of tumor. The pre-existing T790M WT clones that were less sensitive to third-generation EGFR-TKIs were selected and then became the dominant clones under the continuous drug screening [47]. The majority studies identified that the T790M WT clones still retained EGFR activating mutation and were often accompanied by other EGFR independent mechanism [34, 47, 49, 101] (Supplementary Table 1), which suggested T790M loss might not be an independent resistant mechanism and the coexistence mechanisms might be the underlying reasons for resistance.

### BIM deletion polymorphism

Bcl-2-like protein 11 (BIM) is a family member of Bcl-2 protein which promotes apoptosis. A 2903-bp intronic deletion of BIM gene lead to the splice of exon 3 and exon 4, thus BIM isoforms without pro-apoptotic effect are expressed. It was reported BIM deletion polymorphism was detected in 12% of East Asians and associated with primary resistance to first-generation EGFR-TKIs. A recent study revealed that BIM deletion polymorphism could also cause apoptosis resistance to afatinib and AZD9291 in EGFR-mutant cell lines, the combined treatment with histone deacetylase 3 (HDAC3) inhibitor could cope with this resistance by disrupt alternative mRNA splicing of exon 3 to exon 4 [158].

### Loss of activating EGFR

The decrease of EGFR expression [159] and loss of activating EGFR mutation [69] can mediate resistance to AZD9291 and CNX-2006 (a compound of rociletinib) due to loss of target of drugs. The resistant cell lines no longer rely on EGFR signaling for survival, instead they depend on alternative signaling or transformed into other type of histology. Mizuuchi et al. [69] suggested the promising treatment should be a new TKI and EGFR-TKIs is not necessary any more. Besides, navitoclax, a potent inhibitor of Bcl-2 family, may be another therapeutic option [159].

### The therapeutic options of patients with unknown resistance mechanism

Although great effort has made to discover the resistant mechanism of TKIs, there are still patients with unknown resistant mechanism. Evidence showed that 18–30% of the patients who progressed on first-generation EGFR-TKIs lacked clear resistant mechanism [55, 88, 154, 160, 161]. And no identified mechanism was found in more than 22% of cases who progressed on irreversible EGFR-TKIs according to recent studies [18, 19, 35] (Tables 2, 3). What's more, a considerable part of patients refuse the invasive re-biopsy and the tumor samples are in some cases difficult to obtain or insufficient for evaluating molecule status [162, 163]. Noninvasive liquid biopsies based on circulating tumor cells (CTC) or cfDNA analysis overcome the above difficulties and are not affected by spatial and temporal heterogeneity bringing by tissue biopsy [164]. The emerging potential methods of liquid biopsies such as DDPCR or Next Generation Sequencing (NGS) are of high sensitivity and throughput, but more evidences are needed to support its application in clinics.

The ideal situation is that we choose the appropriate target-treatment based on acquired resistant mechanisms, but in most practical cases, clinical failure mode are the main basis of treatment, especially for those without clear resistant mechanisms [165, 166]. There are three clinical modes after progression on EGFR-TKIs. For the patients with slow asymptomatic progression and oligoprogressive disease, the mutant EGFR remains driver gene in most of tumor clones during the disease course and thus continuing TKIs with or without local therapy is recommended. While systemic chemotherapy is fundamental treatment for patients with dramatic symptomatic progression. The present dispute is whether continuation of EGFR-TKIs can be beneficial after resistance to EGFR-TKIs. Two prospective studies may help answering this question. The phase II ASPIRATION study (NCT01310036) [167] aimed at assessing the efficacy and safety of continuation of erlotinib following RECIST progression on first-line erlotinib. Among 176 patients with progressive disease (PD) or death event, 93 continued erlotinib until systemic PD or discontinuation for drug-related adverse events. There was 3.1 months (11.0 vs. 14.1 months) between PFS1 (time to initial progression or death) and PFS2 (time to off-erlotinib PD), which suggested that continuation of EGFR-TKIs was a feasible therapeutic option in patients with slow PD. Another phase III IMPRESS study (NCT01544179) [168] evaluated whether it was beneficial for continuing gefitinib combined with chemotherapy beyond PD in the patients treated with first-line gefitinib. The study did not show any significant differences in PFS between gefitinib group and placebo group (5.4 vs. 5.4 months; HR 0.86, 95% CI 0.65–1.13; *p* = 0.27) and OS of placebo group was longer than gefitinib group (17.2 vs. 14.8 months; HR 1.62, 95% CI 1.05–2.52; *p* = 0.03) though the data were immature, which suggested the standard second-line treatment was chemotherapy after progressed on first-line EGFR-TKIs. Both of the two studies do not consider the resistant mechanisms of patients and thus may give some implications for treatment of patients with unknown resistant mechanisms. The complicated clinical failure mode, however, should be taken into consideration for further investigations. And in fact, the previous treatment, performance status, willingness for treatment of patients, etc. may be more complicated after irreversible TKIs progression, which are also of importance in therapeutic decision-making.

New strategies of EGFR-TKIs in combination with other novel agents regardless of resistant mechanism are currently ongoing to delay or decrease the appearance of resistances. (Table 4).

**Table 4:** The current clinical trials of combination therapies in EGFR-mutant patients in unselected population

Trial	Phase	Irreversible EGFR-TKIs	Combination drugs	Target	Previous treatment	EGFR mutation status
NCT02917993	1/2	AZD9291	INCB039110 (Itacitinib)	JAK1	Prior 1G- or 2G- TKIs	EGFR activating mut+/T790M+
NCT02971501	2	AZD9291	Bevacizumab	VEGFR	Prior 1G- or 2G- TKIs	EGFR activating mut+/T790M+
NCT03050411	1	AZD9291	Apatinib	VEGFR2	EGFR-TKIs (≥6) months	EGFR mut+
NCT02954523	1/2	AZD9291	Dasatinib	Src, c-Kit	No prior EGFR-TKIs	EGFR activating mut+, regardless of T790M
NCT02803203	1/2	AZD9291	Bevacizumab	VEGFR	No prior EGFR-TKIs and VEGF inhibitors	EGFR activating mut+
NCT02520778	1	AZD9291	Navitoclax	Bcl-2	Prior EGFR-TKIs (3G-TKIs allowed in dose escalation phase)	EGFR activating mut+, Regardless of T790M (in dose escalation phase); EGFR activating mut+/T790M+ (dose expansion phase)
NCT02789345	1	AZD9291	Ramucirumab (LY3009806) Necitumumab (LY3012211)	VEGFR EGFR	First-Line 1G- or 2G- TKIs	EGFR activating mut+/T790M+
NCT02454933 (CAURAL)	3	AZD9291	MEDI4736	PD-L1	Prior 1G- or 2G- TKIs	EGFR activating mut+/T790M+
NCT02496663	1	AZD9291	Necitumumab	EGFR	Prior EGFR-TKIs (3G-TKIs allowed in dose escalation phase)	EGFR activating mut+ (T790M-in dose expasion phase)
NCT02503722	1	AZD9291	INK128	mTOR1/2	Prior EGFR-TKIs, (3G-TKIs allowed in dose escalation phase)	EGFR activating mut+ (T790M- in dose expasion phase)
NCT02143466	1	AZD9291	MEDI4736 AZD6094 Selumetinib	PD-L1 C-Met MEK	Prior EGFR-TKIs	EGFR activating mut+/T790M+
NCT02630186	1/2	CO-1686	MPDL3280A	PD-L1	Prior EGFR-TKIs (phase 1); Treatmet naive (phase 2)	EGFR activating mut+ Regardless of T790M
NCT02580708	1/2	CO-1686	Trametinib	MEK	Prior EGFR-TKIs (3G-TKIs allowed in phase 2 group B)	EGFR activating mut+
NCT02335944	1/2	EGF816	INC280	C-Met	Prior EGFR-TKIs (phase 1b and phase 2 group 1) EGFR-TKIs naive (phase 2 Group 2) Treatment naive (phase 2 Group 3)	EGFR activating mut+, De novo T790M(only phase 2 Group 2)
NCT02323126	2	EGF816	Nivolumab	PD-1	Prior EGFR-TKIs	EGFR mut+/T790M+
NCT02716311	2	Afatinib	Cetuximab	EGFR	Treatment naive	EGFR activating mut+/T790M-/exon 20 insertion-
NCT01999985	1	Afatinib	Dasatinib (SRC)	Src, c-Kit	Prior EGFR-TKIs	EGFR activating mut+, De novo T790M mutation
NCT02438722	2/3	Afatinib	Cetuximab	EGFR	Treatment naive	EGFR mut+ (only 19del and 21 L858R)
NCT02364609	1	Afatinib	Pembrolizumab	PD-1	Prior erlotinib	EGFR activating mut+
NCT02191891	1	Afatinib	BI836845	IGF-ligand	Prior EGFR-TKIs or platinum-based chemotherapytreatment (part A) Prior second-generation EGFR TKIs.(part B)	EGFRactivating mut+/T790M- (A) EGFR mut+ (B)

1G-TKIs: first-generation EGFR-TKIs; 2G-TKIs: second generation EGFR-TKIs; 3G-TKIs: third-generation EGFR-TKIs; EGFR mut+: EGFR mutation positive.

## CONCLUSIONS

The irreversible EGFR-TKIs have their own characteristics on inhibition of EGFR signaling when compared with first-generation EGFR-TKIs. The second-generation EGFR-TKIs have broader inhibitory profiles, while the third-generation EGFR-TKIs are more specific to T790M mutation, so the resistant mechanisms have their own features. Some of new resistance mechanisms are introduced, such as C797S/G mutation, T790M loss. And some of mechanisms are absent when compared to reversible EGFR-TKIs, for example HER2 amplification was not reported as resistance mechanism of second-generation EGFR-TKIs. But in short, figuring out whether EGFR-dependent resistance or independent resistance can help the clinician to determine the next therapy. Besides, available data also suggest that > 13% of T790M mutant patients have multiple resistance mechanisms after progression on AZD9291 and CO-1686, so combination and multitargeted therapeutics may be promising strategies to overcome acquired resistance. For the moment, the majority of studies about resistance mechanisms of irreversible EGFR-TKIs are in-vitro, therefore large studies analyzing the biopsy samples of patients are expected.

## SUPPLEMENTARY MATERIALS TABLES




