# Supplementary material for: Mechanisms of resistance to irreversible epidermal growth factor receptor tyrosine kinase inhibitors and therapeutic strategies in non-small cell lung cancer
Source: Oncotarget. 2017 Sep 22;8(52):90557–78. doi: 10.18632/oncotarget.21164 (PMC5685774; doi:10.18632/oncotarget.21164)
Supplement: Supplementary file 1 [file oncotarget-08-90557-s001.pdf]

Mechanisms of resistance to irreversible epidermal growth factor receptor tyrosine kinase inhibitors and therapeutic strategies in non-small cell lung cancer

SUPPLEMENTARY MATERIALS

Supplementary Table 1: EGFR-independent resistance mechanisms of irreversible EGFR-TKIs and potential treatment strategies. See_Supplementary_Table 1
