# Supplementary material for: Mechanisms of resistance to irreversible epidermal growth factor receptor tyrosine kinase inhibitors and therapeutic strategies in non-small cell lung cancer
Source: Oncotarget. 2017 Sep 22;8(52):90557–78. doi: 10.18632/oncotarget.21164 (PMC5685774; doi:10.18632/oncotarget.21164)
Supplement: Supplementary file 2 [file oncotarget-08-90557-s002.docx]

Supplementary Table 1: EGFR-independent resistance mechanisms of irreversible EGFR-TKIs and potential treatment strategies.

| **Resistant mechanisms** | **Drugs** | **Co-existed mechanism** | **Strategies** | **References** |
| --- | --- | --- | --- | --- |
| **Activation of alternative signaling** |  |  |  |  |
| C-met amplification | Afatinib, Dacomitinib | T790M  C-KIT  ERBB3  EMT | Crizotinib (c-Met/ALK inhibitor)/  PHA-665752 (c-Met inhibitor)/  E7050 (c-Met/VEGFR-2 inhibitor)  + 2G-TKIs | [[18](#_ENREF_18), [67](#_ENREF_67)] [[70](#_ENREF_70)][[155](#_ENREF_155)] |
|  | AZD9291, CNX-2006, CO-1686, WZ4002, EGF816 | C797S  ERBB2 amplification  KRAS mutation  PIK3CA mutation  KRAS mutation  Met mutation  CDKN2A mutation  BRAF rearrangement | Crizotinib/  PHA-665752/  ARQ179 (c-Met inhibitor)/  SGX523 (c-Met inhibitor)/  E7050/  JNJ-61186372 (EGFR/Met antibody)+ 3G-TKIs | [[36](#_ENREF_36), [56](#_ENREF_56), [61](#_ENREF_61), [69-71](#_ENREF_69), [101](#_ENREF_101)] |
| HGF overexpression | CL-387,78,  Afatinib,  WZ4002 | - | Anti-HGF neutralizing antibody+ CL-387,785 HGF antagonist NK4+ CL-387,785  SU11274(c-Met inhibitor)+ CL-387,785  Crizotinib+ afatinib,  Crizotinib+ WZ4002 | [[60](#_ENREF_60), [63](#_ENREF_63), [66](#_ENREF_66)] |
| IGF signaling activation |  |  |  |  |
| IGF1R activation  IGF3BP loss  IGFBP3 upregulation | Dacomitinib WZ4002  Afatinib | ERK activation | BMS536924 (IGF-1R/IR inhibitor)+ dacomitinib/WZ4002  PI-103 (PI3K inhibitor)+ dacomitinib/ WZ4002  Recombinant IGFBP3+ dacomitinib  Linsitinib (IGF-1R/IR inhibitor)+ afatinib  AG-1024 (IGF-1R inhibitor)+ WZ4002  BI 836845 (IGF ligand-neutralizing antibody)+ WZ4002 | [[76-78](#_ENREF_76)] |
| HER2 amplification | AZD9291, CO-1686 | C-Met amplification,  CDKN2A mutation,  PI3KCA | - | [[35](#_ENREF_35), [36](#_ENREF_36), [89](#_ENREF_89), [101](#_ENREF_101)] |
| Gas6/AXL activation | CO-1686, AZD9291 | EMT  FGFR1 activation | R428 (AXL inhibitor)+ CO-1686  XL-880 (VEGFR/MET/AXL inhibitor)+ CO-1686 | [[9](#_ENREF_9), [44](#_ENREF_44)] |
| FGFR 1 activation | Afatinib | - | PD173074 (FGFR1/VEGFR2 inhibitor)+ afatinib | [[100](#_ENREF_100)] |
|  | AZD9291 | T790M loss | PD173074  BGJ398 (pan-FGFR inhibitor) | [[48](#_ENREF_48), [49](#_ENREF_49), [101](#_ENREF_101)] |
| IL-6 activation | Afatinib | - | AG490 (JAK2 inhibitor)+ afatinib  Pyridone 6 (Pan-JAK inhibitor)+ afatinib | [[102](#_ENREF_102)] |
| SFK/FAK signaling activation  (YES1 amplification) | AZD9291, afatinib | - | SFK inhibitor (PP2, dasatinib, bosutinib and saracatinib)+ AZD9291,  dasatinib+afatinib  FAK inhibitor (PF573228)+ AZD9291 | [[103](#_ENREF_103), [104](#_ENREF_104)] |
| EPHA2 overexpressed | AZD9291 | - | EPHA2 inhibitor (ALW-II-41-27) | [97] |
| **Alterations of MAPK pathway** |  |  |  |  |
| KRAS gain | Afatinib | - | - | [[35](#_ENREF_35), [36](#_ENREF_36), [101](#_ENREF_101)] |
|  | AZD9291  WZ4002 | - | Selumetinib (MEK1 inhibitor)+ WZ4002 |  |
| KRAS mutation  (KRAS G12A/S, Q61H/K and A146T, G13D) | AZD9291  CO-1686 | PI3KCA mutation  KIT mutation  EGFR T790M/C797S | Selumetinib+ AZD9291  Trametinib (MEK1/2 inhibitor)+ AZD9291 |  |
| NRAS gain | Afatinib | - | Selumetinib+afatinib | [[15](#_ENREF_15), [68](#_ENREF_68)] |
|  | AZD9291, ASP8273 | MAPK1 gain/CRKL gain | Selumetinib+3G-TKIs,  Trametinib+3G-TKIs |  |
| NRAS mutation (Q61K, E63K, G12V and G12R) | WZ4002, AZD9291 | - | Selumetinib |  |
| BRAF mutation(V600E) | AZD9291 | - | Encorafenib(BRAF inhibitor)+ AZD9291 | [[101](#_ENREF_101), [124](#_ENREF_124)] |
| BRAF rearrangement | EGF816 | C-Met amplification | - | [[61](#_ENREF_61)] |
| MAPK1 (EKR2) amplification | WZ4002, AZD929 | EMT  decreased of DUSP6  NRAS gain  CRKL amplification | U0126(MEK inhibitor)+ WZ4002  Trametinib+ WZ4002  CI-1040(MEK inhibitor)+ WZ4002  Selumetinib | [[15](#_ENREF_15), [125](#_ENREF_125)] [[126](#_ENREF_126), [128](#_ENREF_128)] |
| NF1 loss | Afatinib | - | MEK inhibitor+ TKIs | [[127](#_ENREF_127)] |
| Decreased of DUSP6 | Dacomitinib  WZ4002 | IGF1R activatio  MAPK1 amplification | CI-1040+ dacomitinib  CI-1040+ WZ4002 | [[76](#_ENREF_76), [125](#_ENREF_125)] |
| CRKL amplification | AZD9291 | NRAS gain/MAPK1 amplification | Selumetinib | [[15](#_ENREF_15), [114](#_ENREF_114)] |
| **Alterations of AKT pathway** |  |  |  |  |
| PIK3CA mutation（E542K, E545K, E81K） | CO-1686, AZD9291 | C-Met amplification,  EGFR/HER2 amplification | - | [[35](#_ENREF_35), [49](#_ENREF_49), [101](#_ENREF_101)] |
| PTEN deletion | AZD9291 | EGF overexpression | - | [[48](#_ENREF_48)] |
| AKT3 activation | CO-1686, AZD9291 | ERK2 activation | MK-2206 (AKT inhibitor)+CO-1686  GDC-0068 (AKT inhibitor)+CO-1686 | [[9](#_ENREF_9), [48](#_ENREF_48)] |
| **Histologic transformation** |  |  |  |  |
| EMT | Afatinib | MiR-200c downregulated  Acquisition of cancer stem cell-like properties  ERK/AKT activation | Bortezomib (20S proteasome inhibitor) | [[150](#_ENREF_150), [171](#_ENREF_171)] |
|  | CO-1686, WZ4002, CNX-2006 | AKT activation  AXL overexpression  MAPK1 amplification  NF-κB activation  Pin1 and autophagy | MK-2206+CO-1686  GDC-0068+CO-1686  XL-880 (VEGFR/MET/AXL inhibitor)+ CO-1686  R428 (AXL inhibitor)+ CO-1686  U0126+ WZ4002  NF-κB inhibitor (TPCA-1/BEZ-235/bortezomib)+ CNX-2006 | [[9](#_ENREF_9), [126](#_ENREF_126), [151](#_ENREF_151), [152](#_ENREF_152)]. |
| SCLC transformation | Afatinib | - | - | [[172](#_ENREF_172)] |
|  | AZD9291, CO-1686 | T790M loss | Etoposide + platinum | [[47](#_ENREF_47), [48](#_ENREF_48)] |
| T790M loss | AZD9291, CO-1686, EGF816, WZ4002 | SCLC transformation  FGFR-1 activation  EGFR G724S,  EGFR C797S  EGFR amplification,  PIK3CA E545K  C-Met amplification,  HER2 amplification,  KRAS mutation | - | [[47-49](#_ENREF_47), [61](#_ENREF_61), [101](#_ENREF_101)] |
| Loss of activating EGFR | AZD9291, CNX-2006 | C-Met amplification | Navitoclax | [[69](#_ENREF_69), [159](#_ENREF_159)] |
| BIM deletion polymorphism | AZD9291 | - | HDAC3 inhibitor+AZD9291 | [[158](#_ENREF_158)] |

“-”represents there are no co-existed mechanisms or no treatment strategies, 2G-TKIs: second generation EGFR-TKIs; 3G-TKIs: third-generation EGFR-TKIs.
